# Acute Kidney Injury in the Context of COVID-19: An Analysis in Hospitalized Mexican Patients

**DOI:** 10.3390/idr16030034

**Published:** 2024-05-16

**Authors:** Juan Carlos Borrego-Moreno, María Julieta Cárdenas-de Luna, José Carlos Márquez-Castillo, José Manuel Reyes-Ruiz, Juan Fidel Osuna-Ramos, Moisés León-Juárez, Rosa María del Ángel, Adrián Rodríguez-Carlos, Bruno Rivas-Santiago, Carlos Noe Farfan-Morales, Ana Cristina García-Herrera, Luis Adrián De Jesús-González

**Affiliations:** 1 Instituto Mexicano del Seguro Social, Hospital General de Zona # 1, Servicio de Epidemiologia, Zacatecas 98000, Mexico; bogue6@hotmail.com; 2 Instituto Mexicano del Seguro Social, Unidad de Medicina Familiar # 1, Servicio de Medicina Familiar, Guadalupe, Zacatecas 98608, Mexico; july241086@hotmail.com; 3 Instituto Mexicano del Seguro Social, Unidad de Medicina Familiar # 57, Servicio de Medicina Familiar, Zacatecas 98085, Mexico; edu.med.fam.charly34@gmail.com; 4 División de Investigación en Salud, Unidad Médica de Alta Especialidad, Hospital de Especialidades No. 14, Centro Médico Nacional “Adolfo Ruiz Cortines”, Instituto Mexicano del Seguro Social (IMSS), Veracruz 91897, Mexico; jose.reyesr@imss.gob.mx; 5 Facultad de Medicina, Región Veracruz, Universidad Veracruzana (UV), Veracruz 91700, Mexico; 6 Facultad de Medicina, Universidad Autónoma de Sinaloa, Culiacán 80019, Mexico; osunajuanfidel.fm@uas.edu.mx; 7 Laboratorio de Virología Perinatal y Diseño Molecular de Antígenos y Biomarcadores, Departamento de Inmunobioquímica, Instituto Nacional de Perinatología, Mexico City 11000, Mexico; moisesleoninper@gmail.com; 8 Department of Infectomics and Molecular Pathogenesis, Center for Research and Advanced Studies (CINVESTAV-IPN), Mexico City 07360, Mexico; rmangel@cinvestav.mx; 9 Unidad de Investigación Biomédica de Zacatecas, Instituto Mexicano del Seguro Social, Zacatecas 98000, Mexico; rdz.carlos09@hotmail.com (A.R.-C.); rondo_vm@yahoo.com (B.R.-S.); ana.garciaher@imss.gob.mx (A.C.G.-H.); 10 Departamento de Ciencias Naturales, Universidad Autónoma Metropolitana (UAM), Unidad Cuaji-malpa, Mexico City 05348, Mexico; carlos.farfan@cinvestav.mx

**Keywords:** acute kidney injury (AKI), COVID-19, biomarkers in COVID-19

## Abstract

During the COVID-19 pandemic, a considerable proportion of patients developed a severe condition that included respiratory failure, shock, or multiple organ dysfunction. Acute Kidney Injury (AKI) has been recognized as a possible cause of severe COVID-19 development. Given this, this study investigates the occurrence and consequences of AKI in Mexican patients to contribute to better knowledge and management of this problem. **Methods**: Using a retrospective observational cohort methodology, we investigated 313 cases from a cohort of 1019 patients diagnosed with COVID-19 at the IMSS Zacatecas General Hospital of Zone No. 1 in 2020. The prevalence of AKI was determined using the AKIN criteria based on serum creatinine levels and a detailed review of demographic characteristics, medical history, comorbidities, and clinical development. **Results**: The data showed a 25.30% prevalence of AKI among patients infected with severe COVID-19. Remarkably, these patients with AKI exhibited an advanced age (>65 years), arterial hypertension, a higher number of white blood cells during admission and the hospital stay, and elevated levels of C-reactive protein, serum creatinine, and blood urea nitrogen (BUN). Clinically, patients with AKI had signs of prostration, pneumonia, and the requirement for ventilatory assistance when compared to those without AKI. Finally, those diagnosed with AKI and COVID-19 had a 74% death rate. Relative risk analyses indicated that age (>65 years), arterial hypertension, high creatinine levels, endotracheal intubation, and pneumonia are associated with the development of AKI. On the other hand, among the protective factors against AKI, high hemoglobin levels and the consumption of statins during COVID-19 were found. **Conclusions**: The findings of this study underscore the significance of promptly identifying and effectively managing AKI to potentially alleviate the negative consequences of this complication within the Mexican population during COVID-19.

## 1. Introduction

Despite the current prevalence of limited SARS-CoV-2 transmission [1], the enduring implications and enigmas surrounding the advanced phases of coronavirus disease 19 (COVID-19) continue, necessitating further examination and comprehension.

In this regard, the COVID-19 illness has been distinguished by a significant proportion of asymptomatic individuals. However, approximately 6–10% of patients had a severe presentation of the condition, where respiratory failure, shock, or multiple organ dysfunction characterized this severe manifestation [2,3]. Similarly, acute kidney injury (AKI) has been identified as a consequence of developing severe manifestations. Previous studies showed a high proportion (5–23%) of individuals hospitalized for COVID-19 who developed AKI during the second week of hospitalization [3,4,5]. 

On the other hand, in the initial stages of the pandemic, a study reported that 6.7% of individuals diagnosed with COVID-19 experienced AKI, a condition characterized by a sudden decline in kidney function. Interestingly, the mortality rate associated with AKI in these patients was high, reaching 91% [6,7]. Similarly, a study conducted in New York in 2020 found that 9% (*n* = 3235) of patients treated for COVID-19 required renal replacement treatment, with an AKI-associated mortality rate of 45% [3,4,5]. 

Notably, the prevalence and incidence of AKI in patients with COVID-19 varies widely, as reported in studies from different countries. For example, studies from China have indicated a prevalence of AKI in COVID-19 patients ranging from 0% to 36.6% [8,9]. Likewise, studies from the USA and Europe have presented an incidence of 28.6% and 7.7% for AKI, respectively [10]. In Norwegian hospitals, 32.0% of patients experienced this condition [11], whereas in Belgian hospitals, 85.1% of patients developed AKI [12]. Furthermore, other authors noted that in the first 14 days following intensive care unit admission, 76.7% of patients experienced AKI [13]. 

Several factors could explain this wide range. In this sense, the development of AKI in patients with COVID-19 has been associated with different variables, including smoking, increased levels of C-reactive protein (CRP), the extent of lung involvement, high leukocyte count, and the requirement for mechanical ventilation [14,15,16]. In studies conducted in the United States, additional characteristics include male gender, advanced age, African American ethnicity, and comorbidities such as diabetes, hypertension, cardiovascular illnesses, congestive heart failure, and a high body mass index [17,18]. 

It has been reported that the most common comorbidities conferring renal vulnerability to AKI are diabetes mellitus and hypertension, in addition to hyperlipidemia and chronic kidney disease (CKD), present in 40%, 61.4%, 57.1%, and 22.2% of patients with COVID-19 and AKI, respectively [19,20]. However, these data are different because the comorbidities and factors influencing the development of AKI are different for each population. Hence, it is necessary to carry out studies in several geographical regions to improve comparability.

Despite the variability, the high mortality rates associated with AKI in patients are a common denominator in all these studies. Additionally, the death rate associated with COVID-19 is approximately 16.1% among individuals, where sepsis, multiple organ failure, shock, heart failure, arrhythmias, and AKI are identified as the primary contributing factors [21,22]. Therefore, AKI has also been valued as a predictor of mortality and severe infection from COVID-19.

Zhen Li et al. have suggested that kidney injury could be a significant predictor of the severity of COVID-19. This finding underscores the importance of closely monitoring renal function and implementing early therapeutic interventions [19] to reduce the high mortality rates observed in patients with AKI [20,23]. 

Therefore, searching for and monitoring AKI-associated markers suggests a promising strategy. In this sense, a recent investigation underscores the significance of monitoring creatinine levels, as it reveals that patients who experienced mortality exhibited significantly elevated creatinine levels [24]. 

In Mexico, clinical studies have highlighted the lack of a national registry of AKI cases, and clinical follow-up of patients who have experienced AKI episodes is infrequent, restricting the availability of data on disease outcomes [25]. Despite these limitations, the incidence of AKI in patients with COVID-19 has been reported to vary significantly, ranging from 18% to 58.6%. Systemic arterial hypertension (SAH) is a prominent comorbidity in the development of AKI among these patients, with a prevalence of 28.8%-72.5%. On the other hand, an associated mortality rate of 65.5% has been recorded. However, the variability in the results of these studies is considerable, based on small samples ranging from 22 to 266 patients [26,27,28,29]. 

The health sector in Mexico has documented that 23% of COVID-19 mortality is associated with AKI, highlighting the need to identify additional factors that precipitate AKI. This could drive public health initiatives, including national nephrology programs and the creation of departments specialized in kidney diseases, to address this problem [25]. In this context, our retrospective study seeks to examine the factors related to AKI in Mexican individuals affected by COVID-19, admitted to the Mexican Social Security Institute (IMSS) in the city of Zacatecas, highlighting the importance of better understanding and management of the LRA during the pandemic.

## 2. Materials and Methods

### 2.1. Research Design

A retrospective cohort observational study was conducted at the General Hospital of Zone No. 1 of the IMSS Zacatecas in 2020. The current study underwent evaluation by the Local and National Committee for Research and Ethics in Health Research of the IMSS, with registration number R-2021-3301-007. The study received authorization to conduct a retrospective analysis of clinical records. Data were fully anonymized before being accessed, thereby preventing the need for informed consent.

### 2.2. Study Population and Sample

**Figure 1:**
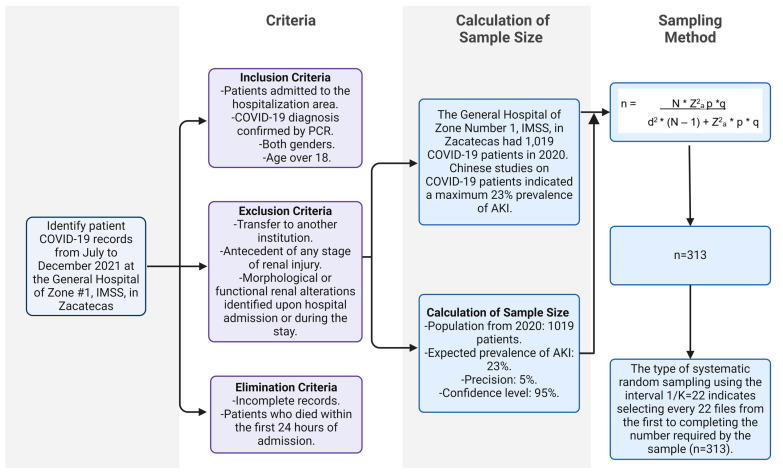
Calculation and selection of study sample.

The study involved a cohort of 1019 patients who were diagnosed at the IMSS with COVID-19 in the year 2020. A sample of 313 cases was selected using a finite population technique to ensure representativeness (Figure 1 and Appendix A). The study enrolled individuals of both genders who were at least 18 years old and had received a confirmed diagnosis of COVID-19 using real-time PCR testing (Logix Smart ABC (Influenza A/B, SARS-CoV-2) Test Kit).

### 2.3. Selection and Randomization

Patients admitted to the hospital were listed consecutively according to the date of their admission. The files were selected for evaluation using a random probabilistic selection method. Clinical cases that did not meet the inclusion criteria (over 18 years of age, confirmed diagnosis of COVID-19, patients treated at the General Hospital of Zone No. 1 of the IMSS Zacatecas and complete clinical records) or that met the exclusion criteria (pregnancy and incomplete clinical records, and CKD) were discarded.

### 2.4. Variables

The primary dependent variable in this study was the incidence of acute renal injury, as determined by the AKIN criteria, using the serum creatinine (SCr) criteria [30]. Independent variables were selected for their clinical relevance and their previously documented association with AKI in the context of severe respiratory infections, including COVID-19 [22,31,32,33,34,35]. These variables include demographic parameters, medical history, comorbidities, laboratory findings, clinical progression, and evaluation of AKI.

### 2.5. Statistical Analysis

The normality of the data was previously evaluated through the Kolmogorov–Smirnov test. Initially, a bivariate analysis was conducted, wherein the categorical variables were compared using the Chi-square test, and a bivariate analysis was performed according to the study of the retrospective cohort. The odds ratio was calculated wherein the categorical variables were compared, identifying relative risk with a confidence interval at 95%, while the quantitative variables were assessed using Student’s T-test. The intragroup analysis used the Kruskal–Wallis method for *p* values, with the Bonferroni adjustment. Furthermore, the Kaplan–Meier survival analysis was utilized to assess the survival rates of patients with and without AKI. All statistical tests were two-sided; a *p*-value < 0.05 was considered statistically significant.

### 2.6. Ethical Considerations

All subjects gave informed consent for inclusion before their participation in the study. Ensuring the confidentiality of the information was consistently upheld following research laws and the overarching principles outlined in the general health law. Additionally, the study was carried out following the Declaration of Helsinki, and the protocol’s methodological, ethical, and research quality was approved by the Local Health Research Committee (IMSS), with the institutional registration number R-2021-3301-007 (16 July 2021).

## 3. Results

### 3.1. Sample Overview

The study cohort comprised 313 patients who were diagnosed with COVID-19. These individuals were categorized into two groups based on the presence or absence of AKI. A prevalence of AKI amounting to 17.25% (*n* = 54) was identified throughout the entire cohort of cases under evaluation.

In the population, the male gender predominated, and the average population age was 60.13 years (SD ± 14.84). However, in the stratification of the patients, it was evident that the individuals with AKI were older (65.39, SD ± 14.51) compared to the patients without AKI (59.03, SD ± 14.7). This variable stood out as a risk factor for the development of AKI during COVID-19 (RR 2.076, CI 1.902, 95% 2.250).

It was discovered that a majority, 86.9% (*n* = 272), of the patients reside in the central region of the state of Zacatecas (Table 1).

In the context of personal and pathological history, it was observed that arterial hypertension is a risk factor for the development of AKI during COVID-19 (RR 1.128, CI 1.014, 95% 1.254) since this showed statistical differences, present in 45.05% of the total population and 59.26% of patients with AKI. Furthermore, in the AKI categories, AKI-I (72.2%) frequency was higher than AKI-II (51.9%) and III (55.6%). On the other hand, the presence of asthma was related to the development of AKI III (*p* = 0.046). (Table 2 and Supplementary Table S1).

Regarding paraclinical (laboratory) characteristics, compared to those without AKI, patients with AKI showed statistical differences in leukocyte values upon admission and during the hospital stay. In the white blood cell count, creatinine, BUN, and CRP differences were also shown. However, high creatinine levels (>1.3 nmol/l) were identified as a risk factor for the development of AKI during COVID-19 (RR 3.076, CI 1.902, 95% 3.250) and, as a protective factor for AKI, high hemoglobin values (RR 0.018, CI 0.0, 95% 0.037) (Table 3 and Supplementary Table S1).

### 3.2. Comparison of Laboratory Data between Groups with AKI and without AKI

In this study, we compare two distinct groups: individuals diagnosed with AKI and those without AKI. Among the cohort of patients, it was observed that 25.30% of them exhibited AKI. Further analysis revealed that 59.26% (*n* = 30) had a preexisting arterial hypertension condition within this subgroup. In contrast, arterial hypertension was identified in only 42.08% of cases without AKI. This disparity was statistically significant, with a *p*-value of 0.021. Additionally, there were variations in the prevalence of COPD history, with rates of 12.96% and 15% (5.79%, *p* = 0.061), correspondingly.

On the other hand, in immunization history, the distribution of individuals with AKI and those without AKI was comparable, with percentages of 18.52% and 16.99%, respectively. Consequently, no statistically significant differences were seen between these two groups (*p* = 0.787), as indicated in Table 2.

When comparing the laboratory parameters of the groups with and without AKI, it was observed that the distribution of leukocytes at admission was 12.38 (±4.85) in the AKI group and 10.68 (±5.29) in the non-AKI group (*p* = 0.030). Furthermore, during their hospitalization, the average leukocyte values were found to be 22.02 (±11.12) in the non-AKI group and 15.03 (±7.64) in the AKI group (*p* = 0.000).

In a similar vein, our study revealed that the observed BUN values in patients diagnosed with AKI had a range of 37.32 (±25.7) compared to 24.02 (±15.61) in non-AKI patients (*p* = 0.000). On the other hand, the mean creatinine values in those with AKI were 1.56 (±1.68), compared to 1.11 (±1) in those without AKI (*p* = 0.009). Similarly, among patients without AKI, the distribution of C-reactive protein levels in AKI cases was 186.24 (±122.65), while it was 135.2 (±107.03) in those without AKI (*p* = 0.002) (Table 3).

### 3.3. Comparison of Clinical Characteristics between Groups with AKI and without AKI

Additionally, we conducted an analysis based on the patient’s clinical presentation. Our findings revealed that within the group of patients diagnosed with AKI, 9.26% (*n* = 5) had a previous history of prostration. In contrast, only 2.7% (*n* = 7) of the patients without AKI reported a similar history. This characteristic demonstrated a significant difference between the two groups (*p* = 0.022). However, other clinical indicators such as fever, dehydration, cough, headache, odynophagia, and myalgia did not exhibit statistical significance (Supplementary Table S1).

Concerning the clinical progression of patients who exhibited AKI, seven individuals (12.96% of the sample) had a documented record of utilizing renin–angiotensin–aldosterone system inhibitors (RCTs). In contrast, 46.3% had a history of non-steroidal anti-inflammatory drug (NSAID) usage before hospitalization (Table 4).

Notably, no statistically significant disparities were observed when comparing this subgroup to the cohort of AKI cases without any reported renal impairment. Additionally, it was shown that among the patients diagnosed with AKI, 50% (*n* = 27) necessitated endotracheal intubation, but only 14.29% (*n* = 37) of those without AKI required this intervention (*p* = 0.000).

In the group of individuals with AKI, 44.44% (*n* = 24) were clinically diagnosed with pneumonia, but only 7.34% of the group without AKI received this diagnosis (*p* < 0.000). Similarly, radiography confirmed the diagnosis of pneumonia in 42.50% of patients with AKI. There was a significant difference in the duration of hospitalization between patients with AKI and those without AKI (*p* < 0.000). The mean length of stay for AKI cases was 12.2 (±8.9) days, whereas for non-AKI patients, it was 8.7 (±7) days (*p* = 0.003). In addition, it was observed that a higher mortality rate of 74.0% was recorded among those with AKI, compared to a mortality rate of 39.77% (*n* = 103, *p* = 0.000) in the group without AKI, as indicated in Table 4.

Finally, statin consumption seems to be associated with a protective factor for the development of AKI (RR 0.824, CI 0.783, 95% 0.868). On the other hand, the risk analysis identified endotracheal intubation (RR 1.542, CI 1.245, 95%, 1.910), pneumonia (RR 1.958, CI 1.400, 95%, 2.738), and death (RR 4.327, CI 2.242, 95%, 8.352) as factors associated with the development of AKI. Furthermore, the presence of pneumonia and death was identified as a risk associated with the development of AKI-II (*p* = 0.000) and AKI-III (*p* = 0.000) (Supplementary Table S1).

### 3.4. Survival Analysis between COVID-19 Patients with AKI and without AKI

Finally, we conduct a survival analysis comparing individuals who develop AKI with those who do not develop AKI during COVID-19.

**Figure 2:**
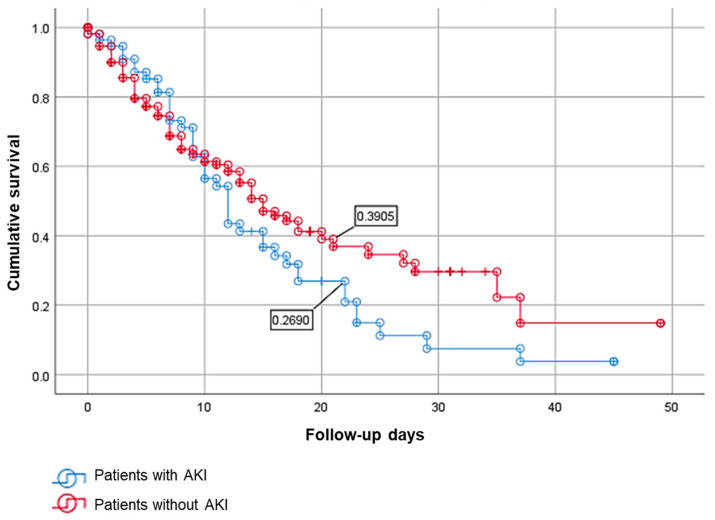
Analysis of survival during the hospital stay of patients with COVID-19 who presented with AKI and without AKI. Kaplan–Meier curve *p*-value of 0.135.

The examination of the Kaplan–Meier curve revealed an apparent elevated death rate among patients who developed AKI during the first 20 days of hospital stay compared to those who did not have AKI, a distinction that continued until 45 days of stay. Nevertheless, the observed result did not achieve statistical significance, with a log-rank *p*-value of 0.135 (Figure 2).

The present analysis comprehensively examines the attributes and progression of individuals admitted to hospitals with COVID-19, underscoring the need to recognize AKI as a potential determinant of unfavorable clinical results.

## 4. Discussion

The literature shows that the occurrence of AKI in individuals diagnosed with COVID-19 exhibits variability, contingent upon the specific population under investigation. In the initial publications, including limited sample sizes, Cheng et al., Zhou et al., Diao et al., and Li et al. documented incidences of AKI ranging from 5% to 23% [4,5,15,19]. 

Nonetheless, our study unveiled a higher prevalence rate of 25.30% for AKI. Recent research indicates even higher AKI rates, which can be ascribed to several factors [12,13]. For instance, multi-organ support, including mechanical ventilation, vasopressor support, and Extracorporeal membrane oxygenation, may play a role in this pattern, which was not observed in our study.

The average age of patients who experienced AKI in our research was 65.39 years (±14.84 years), which aligns with the median age of 63 years reported by Cheng Y. et al. [15]. Regarding gender distribution, it is noteworthy that 57.83% of the participants in our sample were identified as male. This proportion aligns closely with the findings given by Pei, G. et al. and Mohamed, M. M. et al., who reported a value of 52.4% in their respective studies [17,18]. Nevertheless, the observed gender disparity within our sample did not yield statistically significant results.

In contrast, Pelayo J et al. (2020) emphasized the presence of systemic arterial hypertension as a comorbidity linked to AKI, with a prevalence of up to 72% in a series of cases seen in New York [36]. In the present study, hypertension was seen in a comparatively smaller proportion, precisely 59.26%, among patients diagnosed with AKI.

Interestingly, clinical data suggest that arterial hypertension is one of the most prevalent cardiovascular comorbidities in COVID-19 patients, which can exacerbate outcomes and increase the risk of intensive care unit (ICU) admissions [37,38,39]. In Mexico, the prevalence of arterial hypertension among adults is 49.4% (44.0% in women and 55.3% in men), so these data are concerning in terms of the development of AKI during COVID-19 [40]. 

In their study, Gameiro J et al. observed a median serum creatinine level of 1.00 mg/dL [41] while analyzing laboratory data. However, our research findings indicate that the levels observed in the group with AKI were significantly higher, with a mean of 1.56 mg/dL (±1.68) (*p* = 0.009). Furthermore, our findings revealed a C-reactive protein level of 186.24 mg/dL (±122.65), which differs from the 97.1 ± 8.72 mg/dL reported by Gameiro J et al. [41].

In our study, patients with AKI had an admission leukocyte count of 12.38 (4.85) cells/mL, whereas patients without AKI had an admission leukocyte count of 10.68 (5.29) cells/mL (*p* = 0.030). During the hospital stay, these altered leukocyte levels were maintained (*p* = 0.000). Numerous studies have described leukocyte levels at discharge as biomarkers of COVID-19 severity and mortality [42,43,44,45]. Our study links these levels to the development of AKI, a severe COVID-19 complication.

In contrast, our study detected altered blood urea nitrogen (BUN) levels in patients with AKI (*p* = 0.000) as a result of kidney injury. This metabolite has been characterized as a biomarker of mortality among COVID-19 patients [46,47], and in the present study, we also describe it as a biomarker for the onset of AKI.

The analysis of biomarkers during COVID-19 has a critical role in lowering mortality [32,33,48]. Our research has emphasized the significance of several biomarkers (such as increased white blood cell count on admission and during hospitalization and heightened levels of C-reactive protein, serum creatinine, and BUN) that may suggest AKI development. These findings have the potential to change existing therapeutic practices by implementing preventive methods against AKI and, as a result, reduce the associated fatality rates.

On the other hand, Głowacka et al. (2021) analyzed the utilization of angiotensin-converting enzyme inhibitors (ACE-is) and Angiotensin Receptor Blockers (ARBs) in the context of the risk of AKI among patients diagnosed with COVID-19 [6]. Despite the early disputes surrounding this matter, subsequent studies, like the one conducted by our research team, have effectively dismissed any potential relationship. Our stance aligns with the position of The Council on Hypertension of the European Society of Cardiology and the findings presented by Sanchez et al., which advocate against discontinuing the use of ACE-i or ARB in individuals with mild or moderate cases of COVID-19 [49,50]. 

Since these medications can substantially reduce arterial hypertension [51], which our study identified as the primary comorbidity associated with the onset of AKI in patients with COVID-19, their appropriate administration is crucial. By managing this underlying condition, progression to AKI can be prevented, thereby reducing mortality. Consequently, it is imperative to incorporate these medications into the treatment regimen for patients with COVID-19 and the comorbidity of arterial hypertension to enhance clinical outcomes.

According to our data, there is a strong correlation between the severity of the disease and the development of AKI in patients with COVID-19. Our findings reveal a significant mortality rate of 74.0% among those who suffered ARI. The results of this study align with the findings reported by Głowacka, M. et al., whereby they established a correlation between AKI and increased severity and mortality rates among people diagnosed with COVID-19 [6]. Although no statistically significant values were observed in the survival analysis, a decrease was seen in the survival rates of patients diagnosed with AKI and COVID-19.

In Figure 2, a bifurcation in the death trajectories between the groups with and without AKI is readily visible after the first ten days of hospital stay. Interestingly, this pattern of difference is similarly seen in prior investigations, which have identified important symptomatologies contributing to death. Among these are sepsis, which typically appears around day 9–10; liver dysfunction, which typically peaks around day 13.5; and ARDS, which typically occurs around day 10–12. In addition, invasive mechanical ventilation being implemented after ten days of symptom onset, admission to the ICU, and the presence of acute cardiac injuries and AKI (typically between days 15 and 18) are all recognized determinants of respiratory worsening [4,52,53]. It is possible that AKI is playing a role in the observed discrepancy; nevertheless, a more thorough investigation is needed to completely clarify the impact of AKI and other intervening events on the observed mortality.

Relative risk analyses indicated that age (>65 years), arterial hypertension, high creatinine levels, endotracheal intubation, and pneumonia are associated with the development of AKI during COVID-19, variables discussed previously [54]. 

On the other hand, high hemoglobin levels and the consumption of statins during COVID-19 were protective factors associated with the development of AKI. Various studies have linked low hemoglobin levels during COVID-19 with poor outcomes. This has even been associated with other clinical syndromes, such as pulmonary edema based on arterial vasoconstriction and altered alveolocapillary barrier, sideroblastic anemia, endothelitis, vasospastic acrosyndrome, and arteriovenous thromboembolism [55,56]. 

The consumption of statins has been mainly associated with the depletion of intracellular lipids, which can affect different viral stages of both SARS-CoV-2 and other viruses, such as viral entry and replication [57]. One of its effects may be the destabilization of lipid rafts, where viral receptors are anchored, and the decrease in lipids in the endoplasmic reticulum that allows the formation of new viral particles [58]. In addition, it has been found that atorvastatin can prevent the transport of viral proteins to the nucleus by controlling the sequestration of cellular messenger RNA and decreasing viral replication [59,60]. 

On the other hand, statins appear to play a crucial role in modulating the immune response at various levels. Growing evidence has identified statins as potential anti-inflammatory agents. As described above, several cholesterol metabolites and their associated nuclear receptors can regulate the immune system, effects that may prevent the development of AKI [61,62,63]. 

Finally, this study has limitations inherent to its retrospective design, including dependence on existing records and possible biases arising from sample selection. In addition, the evolution in treatment strategies during the study period, the influence of circulating SARS-CoV-2 variants, and the contextual relevance of the data in a specific time frame must be considered. However, its strengths are notable, such as its clinical significance, which is essential for optimizing patient results, its broad database, practical applicability, and its detailed focus on Mexican patients with particular comorbidities. In addition, it contributes to the design of early intervention strategies, which have significant potential to reduce mortality associated with COVID-19.

## 5. Conclusions

In our study, we observed that the incidence of AKI among COVID-19 patients was 25.30%, a figure notably higher than those reported in the international literature. Notably, individuals who succumbed to AKI had discernible characteristics: they were often above 65 years of age, had a medical history dominated by arterial hypertension, exhibited elevated white blood cell counts and CRP levels, and faced a grim clinical trajectory characterized by prostration. Further exacerbating their condition, many had clinically and radiologically confirmed pneumonia, necessitating ventilatory support. Tragically, this confluence of conditions culminated in a shocking % mortality rate of 74% among COVID-19 patients with AKI treated at the General Hospital of Zone No. 1 of the IMSS in Zacatecas, Zacatecas. These findings underscore the pressing need for targeted interventions tailored to the specific risks and challenges the Mexican population faces, ensuring timely medical responses and potentially altering the course of clinical outcomes.

## Figures and Tables

**Table 1:** Sociodemographic data of patients with COVID-19.

Variable	Sample Total (*n* = 313)	Presence of AKI (*n* = 54)	Absence of AKI (*n* = 259)	*p*-Value
Age ^+^				0.004 *
(mean years)	60.13 (±14.84)	65.39 (±14.51)	59.03 (±14.7)	
Gender °				0.710
Female	132 (42.17%)	24 (44.44%)	108 (41.7%)	
Male	181 (57.83%)	30 (55.56%)	151 (58.3%)	
Presence of obesity °	65 (20.77%)	12 (22.22%)	53 (20.46%)	0.772
Zacatecas area °				0.050 *
Center	272 (86.9%)	45 (83.33%)	227 (87.64%)	
Northeast	1 (0.32%)	0 (0%)	1 (0.39%)	
North	1 (0.32%)	0 (0%)	1 (0.39%)	
West	2 (0.64%)	0 (0%)	2 (0.77%)	
South	6 (1.92%)	0 (0%)	6 (2.32%)	
Southeast	31 (9.9%)	9 (16.67%)	22 (8.49%)	

* Statistical significance. ° Values presented in absolute frequency (percentage; Chi 2). ^+^ Mean (Standard deviation). Variables’ normal distribution (Kolmogorov–Smirnov).

**Table 2:** Pathological and non-pathological history of interest of patients with COVID-19.

Variable	Sample Total (*n* = 313)	Presence of AKI (*n* = 54)	Absence of AKI (*n* = 259)	*p*-Value
Smoker	30 (9.58%)	5 (9.26%)	25 (9.65%)	0.929
Arterial Hypertension	141 (45.05%)	32 (59.26%)	109 (42.08%)	0.021 *
Diabetes	105 (33.55%)	18 (33.33%)	87 (33.59%)	0.907
COPD	22 (7.03%)	7 (12.96%)	15 (5.79%)	0.061
Asthma	5 (1.6%)	2 (3.7%)	3 (1.16%)	0.050 *
Immunosuppression	8 (2.56%)	3 (5.56%)	5 (1.93%)	0.175
HIV	1 (0.32%)	0 (0%)	1 (0.39%)	0.125
Cardiovascular diseases	17 (5.43%)	4 (7.41%)	13 (5.02%)	0.647
SARS-CoV-2 vaccine	54 (17.25%)	10 (18.52%)	44 (16.99%)	0.787

* Statistical significance. Values presented in absolute frequency (percentage; Chi 2).

**Table 3:** Laboratory characteristics of patients with COVID-19 upon admission to the hospital and evolution.

Variable	Sample Total (*n* = 313)	Presence of AKI (*n* = 54)	Absence of AKI (259)	*p*-Value
Hemoglobin g/dL				0.055
(mean) ^+^	14.53 (±2.27)	13.99 (±2.4)	14.64 (±2.23)	
Leukocytes upon admission Cells/mL				0.030 *
(mean) ^+^	10.97 (±5.25)	12.38 (±4.85)	10.68 (±5.29)	
Leukocytes Cells/mL during hospitalization				0.000 *
(mean) ^+^	16.46 (±8.9)	22.02 (±11.12)	15.03 (±7.64)	
White blood cell count				0.003 *
Leukocytosis °	118 (37.7%)	23 (42.59%)	95 (36.68%)	
Leukopenia °	144 (46.01%)	23 (42.59%)	121 (46.72%)	
Normal °	51 (16.29%)	8 (14.81%)	43 (16.6%)	
Creatinine nmol/L				0.009 *
(mean) ^+^	1.18 (±1.16)	1.56 (±1.68)	1.11 (±1)	
BUN nmol/L				0.000 *
(mean) ^+^	26.32 (±18.41)	37.32 (±25.7)	24.02 (±15.61)	
C-reactive protein				0.002 *
(mean) ^+^	144 (±111.36)	186.24 (±122.65)	135.2 (±107.03)	

* Statistical significance. ° Values presented in absolute frequency (percentage; Chi 2). ^+^ Mean (Standard deviation; Student’s t). Variables’ normal distribution (Kolmogorov–Smirnov).

**Table 4:** Treatment was indicated during COVID-19 in patients who presented AKI or absence of AKI.

Variable	Sample Total	Presence of AKI	Absence of AKI	*p*-Value
ACE-i	26 (8.31%)	7 (12.96%)	19 (7.34%)	0.173
Statins	6 (1.92%)	0 (0%)	6 (2.32%)	0.259
ARAs	117 (37.38%)	25 (46.3%)	92 (35.52%)	0.137
NSAIDs (Before hospitalization)	39 (12.46%)	7 (12.96%)	32 (12.36%)	0.902
Endotracheal intubation	64 (20.45%)	27 (50%)	37 (14.29%)	0.000 *
Clinical Diagnosis of Pneumonia	43 (13.74%)	24 (44.44%)	19 (7.34%)	0.000 *
Pneumonia (X-ray)	42 (13.42%)	23 (42.59%)	19 (7.34%)	0.000 *
Death	143 (45.69%)	40 (74.07%)	103 (39.77%)	0.000 *
DHS				0.003 *
(mean)	9.25 (±8)	12.2 (±8.9)	8.7 (±7)	

* Statistical significance. Values are presented in absolute frequency (percentage; Chi 2). Variables’ normal distribution (Kolmogorov–Smirnov).

## Data Availability

The data is unavailable due to privacy or ethical restrictions.

## Supplementary Materials

The following supporting information can be downloaded at: https://www.mdpi.com/article/10.3390/idr16030034/s1.

## Appendix A

Patient Selection Methodology: The selection of 313 patients from a larger cohort was conducted with a focus on data completeness and adherence to AKIN criteria for AKI diagnosis. This was in accordance with finite population correction principles to ensure a representative sample.

Statistical Power Analysis: Our initial power analysis, based on expected AKI prevalence and effect size, indicated that our sample size was sufficient. This is supported by the prevalence rates reported in the literature, which vary significantly:

- Bashir et al. (2022) found a 12.7% prevalence in their study, highlighting the lower end of the spectrum [14].
- Morosini et al. (2021) observed a prevalence of 37.14%, demonstrating the higher variability in different patient populations [64].
- Wen et al. (2020) reported a 41% prevalence in a subset of severe and critical patients, which is particularly relevant to our study’s focus on hospitalized patients [65].

Conclusion: Appendix A will conclude by affirming the methodological soundness of our approach and the statistical power of our study, with detailed references to the literature that support our prevalent findings and sample size determination.
