# Supplementary material for: Acute Kidney Injury in the Context of COVID-19: An Analysis in Hospitalized Mexican Patients
Source: Infect Dis Rep. 2024 May 16;16(3):458–71. doi: 10.3390/idr16030034 (PMC11130795; doi:10.3390/idr16030034)
Supplement: Supplementary file 1 [file idr-16-00034-s001.zip › idr-2850602-supplementary.pdf]

AKI risk factors

	AKI				AKI categories						AKI relative risk			intragroups p value			
	NO (n=259)		AKI (n=54)		I (n=18)		II (n=27)		III (n=9)								
	Frecuencies	%	Frecuencies	%	Frecuencies	%	Frecuencies	%	Frecuencies	%	RR	CI	95%	AKI I (A)	AKI II (B)	AKI III (C)	NO AKI (D)
**Sociodemoghrapics**																	
Age, years (Media, SD)	59.0	14.8	65.4	14.5	62.6	13.9	66.2	15.0	69.6	13.5	2.076	1.902	2.250				
Sex																	
Female	108	41.7%	24	44.4%	9	50.0%	12	44.4%	3	33.3%							
Male	151	58.3%	30	55.6%	9	50.0%	15	55.6%	6	66.7%							
**Ocupation**																	
Employe	127	49.0%	24	44.4%	8	44.4%	14	51.9%	2	22.2%							
Home tasks	60	23.2%	13	24.1%	5	27.8%	6	22.2%	2	22.2%							
retired	43	16.6%	8	14.8%	3	16.7%	3	11.1%	2	22.2%							
Unemploye	29	11.2%	9	16.7%	2	11.1%	4	14.8%	3	33.3%							
**non-pathological history**																	
Days between simptoms and atention (Media, SD)	4.4	3.4	3.9	2.9	4.7	4.0	3.5	2.2	3.3	1.8							
Days between simptoms and hospitalization (Media, SD)	5.7	4.0	5.4	3.6	6.5	4.9	5.0	2.8	4.7	2.0							
First atemtion as non severe and posterior hospitalization as severe case	21	8.1%	6	11.1%	2	11.1%	3	11.1%	1	11.1%							
Previews contact whit covid case	49	18.9%	6	11.1%	3	16.7%	3	11.1%	0	0.0%							
Vaccination	44	17.0%	10	18.5%	3	16.7%	5	18.5%	2	22.2%							
**Comorbidities**																	
Pulmonary obstructive chronic disease	15	5.8%	7	13.0%	3	16.7%	4	14.8%	0	0.0%							
Diabetes	87	33.6%	18	33.3%	7	38.9%	8	29.6%	3	33.3%							
Hypertension	109	42.1%	32	59.3%	13	72.2%	14	51.9%	5	55.6%	1.128	1.014	1.254				
Cardiovascular disease	13	5.0%	4	7.4%	2	11.1%	2	7.4%	0	0.0%							
Ashtma	3	1.2%	2	3.7%	0	0.0%	1	3.7%	1	11.1%						D(0.046)	
Immune depression	5	1.9%	3	5.6%	1	5.6%	2	7.4%	0	0.0%							
Smoking	25	9.7%	5	9.3%	2	11.1%	3	11.1%	0	0.0%							
Obesity	53	20.5%	12	22.2%	1	5.6%	10	37.0%	1	11.1%							
HIV	1	0.4%	0	0.0%	0	0.0%	0	0.0%	0	0.0%							
Other comorbidities	29	11.2%	3	5.6%	2	11.1%	0	0.0%	1	11.1%							
**Sintomatology**																	
Suden disease	65	25.1%	16	29.6%	7	38.9%	7	25.9%	2	22.2%							
Fever	158	61.0%	35	64.8%	10	55.6%	18	66.7%	7	77.8%							
Cofing	196	75.7%	39	72.2%	11	61.1%	20	74.1%	8	88.9%							
Head ache	163	62.9%	33	61.1%	8	44.4%	19	70.4%	6	66.7%							
Odynophagia	95	36.7%	17	31.5%	6	33.3%	8	29.6%	3	33.3%							
poor general condition	124	47.9%	26	48.1%	6	33.3%	15	55.6%	5	55.6%							
Myalgia	160	61.8%	34	63.0%	13	72.2%	17	63.0%	4	44.4%							
Arthralgies	151	58.3%	32	59.3%	12	66.7%	16	59.3%	4	44.4%							
Prostration	7	2.7%	5	9.3%	1	5.6%	3	11.1%	1	11.1%							
Rhinorrhea	43	16.6%	6	11.1%	3	16.7%	2	7.4%	1	11.1%							
Chill	113	43.6%	20	37.0%	8	44.4%	10	37.0%	2	22.2%							
Abdominal pain	36	13.9%	5	9.3%	3	16.7%	1	3.7%	1	11.1%							
Conjunctivitis	14	5.4%	2	3.7%	1	5.6%	1	3.7%	0	0.0%							
Dyspnoea	193	74.5%	40	74.1%	13	72.2%	20	74.1%	7	77.8%							
Cyanosis	12	4.6%	5	9.3%	3	16.7%	2	7.4%	0	0.0%							
Diarrhea	44	17.0%	10	18.5%	3	16.7%	5	18.5%	2	22.2%							
Chest_pain	98	37.8%	19	35.2%	7	38.9%	8	29.6%	4	44.4%							
Polypnea	12	4.6%	5	9.3%	3	16.7%	2	7.4%	0	0.0%							
Coryzza	2	0.8%	2	3.7%	1	5.6%	0	0.0%	1	11.1%						D(0.011)	
Anosmia	39	15.1%	8	14.8%	4	22.2%	3	11.1%	1	11.1%							
Dysgeusia	39	15.1%	7	13.0%	2	11.1%	3	11.1%	2	22.2%							
Dehydration	19	7.3%	7	13.0%	3	16.7%	3	11.1%	1	11.1%							

**Therapeutic indication of drugs with renal activity**															
angiotensin-converting-enzyme (ACE)	19	7.3%	7	13.0%	4	22.2%	2	7.4%	1	11.1%					
Estatins	6	2.3%	0	0.0%	0	0.0%	0	0.0%	0	0.0%	0.824	0.783	0.868		
inhibitors and/or angiotensin-receptor blockers (ARAs),	92	35.5%	25	46.3%	10	55.6%	10	37.0%	5	55.6%					
nonsteroidal anti-inflammatory drugs (AINEs)	32	12.4%	7	13.0%	1	5.6%	4	14.8%	2	22.2%					
**Laboratories**															
Leukocytes 103 Céls/mL (Media, SD)	10.7	5.3	12.4	4.8	11.9	4.8	12.8	4.7	12.0	5.7					
Creatinine mmol/l (Media, SD)	0.8	0.2	2.7	2.2	1.6	0.1	2.3	0.5	6.0	4.0	3.076	1.902	3.250		
Urea mmol/l (Media, SD)	24.0	15.6	37.3	25.7	31.7	13.5	32.7	20.8	62.3	41.6					
Hemoglobin g/dl (Media, SD)	14.6	2.2	14.0	2.4	14.0	2.7	14.4	2.2	12.8	2.2	0.018	0.000	0.037		
C-reactive protein mg/dl (Media, SD)	135.2	107.0	186.2	122.6	186.9	126.3	176.9	126.4	212.9	112.9					
**Clinical severity**															
Endotracheal intubation	37	14.3%	27	50.0%	5	27.8%	16	59.3%	6	66.7%	1.542	1.245	1.910		
X-ray pneumonia	19	7.3%	23	42.6%	4	22.2%	13	48.1%	6	66.7%	1.958	1.400	2.738	D(0.000)	D(0.000)
Days stay	8.6	7.8	12.2	8.9	12.3	10.4	12.7	9.0	10.9	6.0					
Flu like disease	66	25.5%	14	25.9%	5	27.8%	7	25.9%	2	22.2%					
Severe respiratory disease	193	74.5%	40	74.1%	13	72.2%	20	74.1%	7	77.8%					
Decease	103	39.8%	40	74.1%	6	33.3%	25	92.6%	9	100.0%	4.327	2.242	8.352	D(0.000)	D(0.000)
Clinical improvement	156	60.2%	14	25.9%	12	66.7%	2	7.4%	0	0.0%					

SD; Standard deviation. Statistical parameters: Relative Risk CI 95% Followed cohort. For P values intragroups was used Kruskal Wallis method, whit bonferronis adjustment. For numeric variables Linear regretion where calculated
